# Diversity of *Stomylotrema* spp. in the Mexican tropical lowlands: the case of *Stomylotrema bijugum* and *Stomylotrema vicarium* (Digenea: Stomylotrematidae), parasites of aquatic and passerine birds

**DOI:** 10.1017/S0031182025100413

**Published:** 2025-06

**Authors:** Marcelo Tonatiuh González-García, Valerie Pérez-Mancilla, César A. Ríos-Muñoz, Ana Lucia Sereno-Uribe, Martín García-Varela, Mirza P. Ortega-Olivares

**Affiliations:** 1 Departamento de Zoología, Instituto de Biología, Universidad Nacional Autónoma de México, Ciudad de México, México; 2 Posgrado en Ciencias Biológicas, Universidad Nacional Autónoma de México, Ciudad de México, México; 3 Facultad de Ciencias, Universidad Nacional Autónoma de México, Ciudad de México, México

**Keywords:** birds, haplotype network, LDA, Mexico, new records, PCA, phylogeny, *Stomylotrema*

## Abstract

Distinguishing between *Stomylotrema bijugum* and *S. vicarium* is challenging due to their phenotypic plasticity. In this study, adult specimens were recovered from 9 host species in the Mexican tropical lowlands. To explore the morphological differences, 32 morphological characteristics were evaluated in 54 specimens. Linear discriminant analysis provided enough evidence to differentiate the 2 species. Additionally, a principal component analysis (PCA) was performed for each species. The PCA of *S. bijugum* revealed 3 groups separately corresponding to specimens from the 3 hosts, suggesting host-induced phenotypic plasticity, whereas the PCA of *S. vicarium* revealed that the specimens from 3 host species were clustered together, indicating morphometric homogeneity. To confirm the morphological differences between the 2 species of *Stomylotrema*, we sequenced 2 molecular markers: the D1–D3 domains of the large subunit (LSU) from nuclear DNA and nicotinamide adenine dinucleotide dehydrogenase subunit 1 (*Nad1*) from mitochondrial DNA. Sequences of the LSU were aligned and compared with the LSU sequences of other congeneric species available in GenBank. Phylogenetic analyses supported the monophyly of *Stomylotrema*, with 2 main subclades that corresponded to *S. bijugum* and *S. vicarium*. A haplotype network was predicted with 25 *Nad1* sequences, revealing the presence of 2 clusters representing the 2 species separated from each other by 98 substitutions. The current studies on *S. bijugum* and *S. vicarium* revealed new hosts and geographical regions in the Americas, suggesting that both species addressed in the current study can complete their life cycle in the Neotropical region of Mexico.

## Graphical abstract

**Figure:**
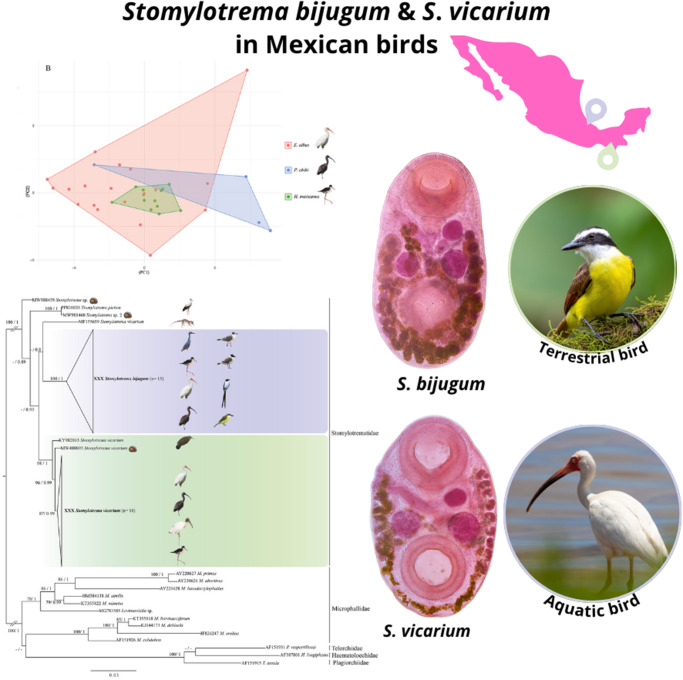

## Introduction

Members of the family Stomylotrematidae Poche, 1926 are endoparasites that are globally distributed and parasitize the digestive tract, caeca, bursa of Fabricii or cloaca of birds. Currently, the family includes 3 genera: *Stomylotrema* Loos, 1900, *Laterotrema* Semenov, 1928 and *Srivastavatrema* Singh, 1962 (Lotz and Font, 2008). The *Stomylotrema* genus represents the most diverse group within the family, with 17 known species. These species are morphologically characterized by the following features: broadly oval body; large, round, terminal oral sucker; well-developed, round ventral sucker; prepharynx short; well-developed pharynx; intestinal bifurcation in the middle third of the body; paired caeca extending near the posterior end of the body; paired, symmetrical testes; presence of a cirrus sac; marginal genital pore at the level of the ventral sucker; submedian, equatorial ovary; well-developed uterus; operculated eggs and presence of Laurer’s canal (Macko et al., 1999; Lotz and Font, 2008; Lunaschi and Drago, 2009; Pinto et al., 2015). In the Americas, 7 species of *Stomylotrema* have been recorded: *S. bijugum* Braun, 1901; *S. fastosum* Braun, 1901; *S. gratiosus* Travassos, 1922; *S. perpastum* Braun, 1902; *S. tagax* Braun, 1901; *S. ucremium* Brenes, Arroyo and Muñoz, 1966 and *S. vicarium*, Braun, 1901 (Szidat, 1964; Ostrowski, 1978; Macko et al., 1999; Pinto et al., 2015). Among these, *S. vicarium* has the widest distribution range in the Americas, extending from the central USA and Cuba to Brazil and Argentina. It has been reported as a parasite of birds from the families Accipitridae, Ardeidae, Ciconiidae, Podicipedidae, Laridae and Threskiornithidae (Macko et al., 1999; Lunaschi and Drago, 2009; Pinto et al., 2015). Macko et al. (1999) performed a morphometric comparison between specimens of *S. bijugum* and *S. vicarium* recovered fromvarious definitive hosts and reported that both species exhibit significant phenotypic plasticity in all metric characteristics, such as the size of the suckers, pharynx, ovary, testes and cirrus sac. Additionally, Pinto et al. (2015) mentioned the complexity of the species limits in *Stomylotrema*. This complexity is because most taxonomic descriptions are based on single adult specimens.

As part of our long-term studies on the biodiversity of helminth parasites of aquatic and passerine birds, digeneans belonging to *Stomylotrema* spp. were recovered from the intestines and cloaca of 9 bird species from 4 localities in the Mexican tropical lowlands. We performed extensive sampling, which allowed us to evaluate the morphology of 2 species, *S. bijugum* and *S. vicarium.* The objectives of the present study were as follows: (1) to provide a revised morphological description of *S. bijugum* and *S. vicarium* from new adult specimens collected from Mexico; (2) to compare morphological and molecular characteristics to investigate the phenotypic plasticity of *S. bijugum* and *S. vicarium* recovered from 9 host species; (3) to generate a haplotype network of the *S. bijugum* and *S. vicarium* specimens by using sequences of nicotinamide adenine dinucleotide dehydrogenase subunit 1 (*Nad1*) from mitochondrial DNA and (4) to test the phylogenetic affinities of *S. bijugum* and *S. vicarium* by using sequences of the D1–D3 domains of the large subunit (LSU) from nuclear ribosomal DNA (rDNA).

## Materials and methods

### Specimen collection and morphological analyses

**Figure 1.**
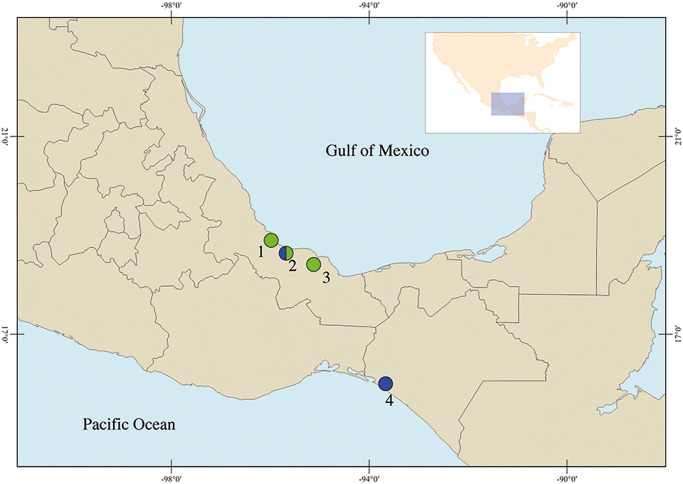
Sampling collection in Mexico. Veracruz: (1) Los Chivos; (2) Tlacotalpan; (3) Catemaco. Chiapas: (4) La Polka. The colours represent the species of *Stomylotrema* spp. recovered: in blue *S. bijugum* and in green *S. vicarium.*

Between 2011 and 2023, 9 bird species belonging to 4 orders from 6 families were collected in 4 localities from Mexican tropical lowlands: *Himantopus mexicanus* (Müller) (Charadriiformes: Recurvirostridae); *Leucophaeus atricilla* (L.) and *L. pipixcan* (Wagler) (Charadriiformes: Laridae); *Mycteria americana* (L.) (Ciconiiformes: Ciconiidae); *Nyctanassa violacea* (L.) (Pelecaniformes: Ardeidae); *Eudocimus albus* (L.) and *Plegadis chihi* (Vieillot) (Pelecaniformes: Threskiornithidae); and *Pitangus sulphuratus* (L.) and *Tyrannus savana* (Daudin) (Passeriformes: Tyrannidae) (Figure 1; Table 1). Birds were identified based on morphological characteristics using field guides for the region (Peterson and Chalif, 1989; Howell and Webb, 1995; Van Perlo, 2006), and the nomenclature follows the American Ornithologists’ Union (1996) until the 65th update (Chesser et al., 2024). Following the capture of the hosts, the digestive tract was removed from the body cavity of each bird and examined under a stereoscopic microscope. The digeneans were washed in 0.75% saline solution, relaxed with hot distilled water and preserved in 70% ethanol for the analyses.

**Table 1.** Taxa used in the present study

					GenBank accession numbers		
Species	Host species	Family host	Infection site	Locality	LSU	*Nad1*	Source
*Stomylotrema bijugum*	*Eudocimus albus*	Threskiornithidae	Intestine (A)	Mexico (2)	**PV700558–PV700561**	**PV738124–PV738127**	**This study**
*Stomylotrema bijugum*	*Himantopus mexicanus*	Recurvitrostridae	Cloaca (A)	Mexico (2)	**PV700562–PV700564**	**PV738130–PV738132**	**This study**
*Stomylotrema bijugum*	*Leucophaeus pipixcan*	Laridae	Cloaca (A)	Mexico (2)	**PV700565–PV700566**	**PV738137–PV738138**	**This study**
*Stomylotrema bijugum*	*Leucophaeus atricilla*	Laridae	Cloaca (A)	Mexico (4)	**PV700567**	**PV738139**	**This study**
*Stomylotrema bijugum*	*Nyctanassa violacea*	Ardeidae	Gizzard (M)	Mexico (2)	**PV700568–PV700569**	**PV738128–PV738129**	**This study**
*Stomylotrema bijugum*	*Pitangus sulphuratus*	Tyrannidae	Cloaca (A)	Mexico (2)	**PV700570**	**PV738135**	**This study**
*Stomylotrema bijugum*	*Plegadis chihi*	Threskiornithidae	Cloaca (A)	Mexico (2)	**PV700571**	**PV738133–PV738134**	**This study**
*Stomylotrema bijugum*	*Tyrannus savana*	Tyrannidae	Cloaca (A)	Mexico (2)	**PV700572**	**PV738136**	**This study**
*Stomylotrema vicarium*	*Eudocimus albus*	Threskiornithidae	Intestine (A)	Mexico (1)	**PV700573**	**PV738140**	**This study**
*Stomylotrema vicarium*	*Eudocimus albus*	Threskiornithidae	Intestine (A)	Mexico (2)	**PV700574–PV700576**	**PV738141**	**This study**
*Stomylotrema vicarium*	*Eudocimus albus*	Threskiornithidae	Intestine (A)	Mexico (3)	**PV700577**	**–**	**This study**
*Stomylotrema vicarium*	*Himantopus mexicanus*	Recurvitrostridae	Cloaca (A)	Mexico (2)	**PV700578–PV700580**	**PV738145–PV738147**	**This study**
*Stomylotrema vicarium*	*Mycteria americana*	Ciconiidae	Gizzard (M)	Mexico (2)	**PV700581**	**–**	**This study**
*Stomylotrema vicarium*	*Plegadis chihi*	Threskiornithidae	Cloaca (A)	Mexico (2)	**PV700582–PV700587**	**PV738142–PV738144**	**This study**
*Stomylotrema vicarium*	*Pomacea americanista*	Ampullariidae	Digestive gland (C)	Argentina	MW480895		Unpublished
*Stomylotrema vicarium*	*Sclerurus mexicanus*	Furnariidae	Intestine (A)	Peru	KY982863		Kanarek et al., 2017
*Stomylotrema vicarium*	*Philander opossum*	Didelphidae	Intestine (A)	Mexico	MF155659		Ramirez-Cañas et al., 2019
*Stomylotrema pictum*	*Ciconia ciconia*	Cinoniidae	(A)	Turkey	PP830835		Unpublished
*Stomylotrema* sp. 1				Egypt	MW988459		Unpublished
*Stomylotrema* sp. 2				Egypt	MW988460		Unpublished
*Maritrema brevisacciferum*	*Caridina indistincta*	Atyidae	(M)	Australia	KT355818		Kudlai et al., 2015
*Maritrema deblocki*	*Anas platyrhynchos*	Anatidae	(A)	New Zealand	KJ144173		Presswell et al., 2014
*Maritrema eroliae*	*Clypeomorus bifasciatus*	Cerithiidae	(C)	Kuwait	JF826247		Al-Kandari et al., 2011
*Maritrema subdolum*	*Tringa erythropus*	Scolopacidae	(A)	Ukraine	AF151926		Tkach et al., 2000
*Microphallus abortivus*	*Hydrobia ulvae*	Hydrobiinae	(C)	United Kingdom	AY220626		Tkach et al., 2003
*Microphallus basodactylophallus*	*Oryzomys palustris*	Cricetidae	(A)	USA	AY220628		Tkach et al., 2003
*Microphallus primas*	*Hydrobia ulvae*	Hydrobiinae	(C)	United Kingdom	AY220627		Tkach et al., 2003
*Microphallus minutus*	*Cherax dispar*	Parastacidae	(M)	Australia	KT355822		Kudai et al., 2015
*Microphallus similis*	*Larus schistisagus*	Laridae	(A)	Russia	HM584138		Galaktionov et al., 2012
*Levinseniella* sp.	*Somateria mollissima*	Anatidae	(A)	Russia	MG783585		Galaktionov and Blasco-Costa, 2018
*Haematoloechus longiplexus*	*Rana catesbeiana*	Ranidae	(A)	USA	AF387801		Snyder and Tkach, 2001
*Plagiorchis vespertilionis*	*Myotis daubentoni*	Vespertilionidae	(A)	Ukraine	AF151931		Tkach et al., 2000
*Telorchis assula*	*Natrix natrix*	Colubridae	(A)	Ukraine	AF151915		Tkach et al., 1999

Localities: Veracruz: (1) Los Chivos. (2) Tlacotalpan. (3) Catemaco. Chiapas: (4) La Polka (localities in parentheses correspond with Figure 1), (M) metacercarie, (A) adult. Sequences in bold were generated in the current study.

The specimens were stained with Mayer’s paracarmine (Merck, Darmstadt, Germany) and mounted on permanent slides with Canada balsam. Digeneans were identified according to Macko et al. (1999) and following the original descriptions. Specimens were photographed and measured using a Leica DM 1000 LED compound microscope (Leica Microsystems CMS GmbH, Wetzlar, Germany); measurements are reported in micrometres (μm). Internal morphological features were illustrated using a drawing tube attached to a Leica MC120HD microscope. Drawings were made using Adobe Illustrator 27.9 (Adobe, Inc., San Jose, CA, USA). The specimens were deposited in the Colección Nacional de Helmintos (CNHE), Instituto de Biología, Universidad Nacional Autónoma de México (UNAM), Mexico City.

### Morphometric analyses

**Figure 2.**
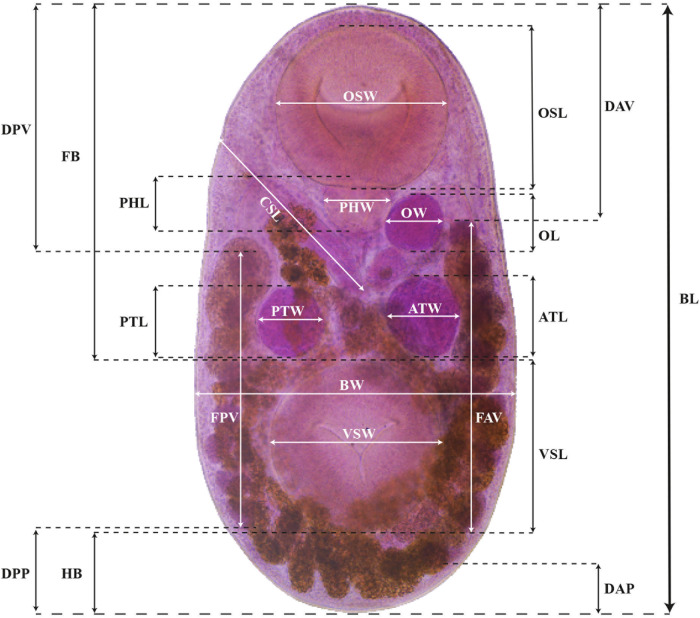
Photomicrograph of *Stomylotrema bijugum*, showing the morphological characters measured. Abbreviations as referred to in the text.

A total of 54 mature adult individuals, 36 of *S. vicarium* and 18 from *S. bijugum*, were analysed. We selected 32 morphological characters (BL, body length; BW, maximum body width; HB, hindbody; FB, forebody; OSL, oral sucker length; OSW, oral sucker width; VSL, ventral sucker length; VSW, ventral sucker width; PHL, pharynx length; PHW, pharynx width; CSL, cirrus sac length; CSW, cirrus sac width; PTL, poral testis length; PTW, poral testis width; ATL, aporal testis length; ATW, aporal testis width; OL, ovary length; OW, ovary width; FPV, field poral vitelline follicles; FAV, field aporal vitelline follicles; DPV, distance poral vitelline to anterior margin; DPP, distance poral vitelline to posterior margin; DAV, distance aporal vitelline to anterior margin; DAP, distance aporal vitelline to posterior margin; R(BL/VSL), ratio (BL/VSL); R(VSW/OSW), ratio (VSW/OSW); R(OSL/PHL), ratio (OSL/PHL); R(OSL/CSL), ratio (OSL/CSL); R(OSW/PHW), ratio (OSW/PHW); AVTW, average testes width; R(MTW/OW), ratio (MTW/OW); and R(CSW/OW), ratio (CSW/OW) (Figure 2). These measures were used in a linear discriminant analysis (LDA) by using a discrimination function that calculates the combination of a minimum number of characters necessary to separate both species sampled. In addition, a principal component analysis (PCA) was implemented to explore the morphological variation of each species analysed. These analyses were run using the ‘stats’ 3.6.2 library in R 4.1.2 (R Core Team, 2022).

### DNA extraction, amplification and sequencing

A total of 31 specimens of *Stomylotrema* spp. were analysed. The genomic DNA was isolated from each specimen, following the protocol described by González-García et al. (2020). The LSU of the nuclear rDNA and *Nad1* were amplified using polymerase chain reaction (PCR). The LSU amplifications used forward primers 391 5’-AGCGGAGGAAAAGAAACTAA-3’ and reverse primers 536 (5’-CAGCTATCCTGAGGGAAAC-3’ (Stock et al., 2001; García-Varela and Nadler, 2005). Additionally, the *Nad1* was amplified using forward NDJ11F 5’-AGATTCGTAAGGGGCCTAATA-3’ (Morgan and Blair, 1998) and reverse NDJ2AR 5’-CTTCAGCCTCAGCATAAT-3’ primers (Kostadinova et al., 2003) PCR (final volume 25 μL) containing 2 μL of each primer (10 pmol μL^−1^), 2.5 μL of 10× buffer, 1.5 μL of 2 mM MgCl_2_, 2 μL of genomic DNA and 1 U of Taq DNA polymerase (Platinum Taq, Invitrogen Corporation, California, USA). PCR cycling parameters include denaturation at 94°C for 3 min, followed by 35 cycles of 94°C for 1 min; annealing at 50°C for LSU and 40°C for *Nad1* for a min; and extension at 72°C for 1 min, followed by a post-amplification incubation at 72°C for 7 min. Sequencing reactions were performed with the initial primers plus 2 internal primers 503, 5’-CCTTGGTCCGTGTTTCAAGACG-3’, and 504, 5’-CGTCTTGAAACACGGACTAAGG-3’ for LSU (García-Varela and Nadler, 2005) using ABI Big Dye (Applied Biosystems, Boston, MA, USA) terminator sequencing chemistry. Reaction products were separated and detected using an ABI 3730 capillary DNA sequencer. Contigs were assembled and base-calling differences resolved using CodonCode Alligner version 12.0.1 (CodonCode Corporation, Dedham, MA, USA). Sequences were deposited in the GenBank database (Table 1).

### Alignment, phylogenetic analyses, genetic divergence and haplotype network

Thirty-one new sequences of LSU were aligned with other sequences identified as *Stomylotrema* spp., 1 sequence of *Stomylotrema pictum* Creplin, 1837, 3 sequences identified as *S. vicarium* (KY982863, MW480895 and MF155659), plus 2 unidentified sequences of *Stomylotrema* sp. (MW988459 and MW988460), plus 10 sequences representing species from genera *Maritrema, Microphallus* and *Levinseniella* from the family Microphallidae and 3 sequences of Haematoloechidae, Plagiorchiidae and Telorchiidae were used as outgroups (Table 1). Sequences were aligned using the software SeaView version 4.0 (Gouy et al., 2010) with default parameters and adjusted with the Mesquite program (Maddison and Maddison, 2025). The alignment consisted of 50 sequences with 1338 nucleotides. The nucleotide substitution model was obtained using jModelTest v2.1.7 (Darriba et al., 2012), and the selection was based on the Akaike information criterion. The best model selected was GTR + I + G. Phylogenetic trees were constructed using maximum likelihood (ML) and Bayesian inference (BI) methods, using the online interface Cyberinfrastructure for Phylogenetic Research (CIPRES) Science Gateway version 3.3 (Miller et al., 2010). The ML analysis was carried on with RAxML version 7.0.4 (Silvestro and Michalak, 2012) and was run with 1000 bootstrap replicates. BI analysis was inferred with MrBayes version 3.2.7 (Ronquist et al., 2012) and included 2 simultaneous runs of Markov Chain Monte Carlo for 10 million generations, sampling every 1000 generations, with a heating parameter value of 25% ‘burn-in’ %. Phylogenetic trees were drawn and edited using the FigTree program v. 1.4.2. (Rambaut, 2012). The genetic divergences among taxa were estimated using *p* distances with the program MEGA version 6.0 (Tamura et al., 2013). To examine the haplotype frequency and relationships among the specimens of *S. vicarium* and *S. bijugum* recovered from 9 host species, a haplotype network was built with 25 *Nad1* sequences by using the TCS algorithm (Clement et al., 2002) implemented in PopART software (Leigh and Bryant, 2015).

## Results

### Morphological identification

Taxonomic summary

Class Trematoda Rudolphi, 1808

Order Plagiochiida La Rue, 1957

Family Stomylotrematidae Poche, 1926

Genus *Stomylotrema* Looss, 1900

*Stomylotrema bijugum* Braun, 1901

*Site of infection*: Cloaca

*Type host*: (1) Charadriiformes: Recurvirostridae: *Himantopus mexicanus* (Müller)

*Type locality*: (1) Brazil (unspecified locality).

*Other localities*: (2) Cuba; (3, 4) Mexico.

Records: Adult specimens, 1. Braun (1901); 2. Macko et al. (1999) 3. CNHE 12081; 4. This study.

*Other definitive hosts*: **Charadriiformes: Jacanidae**: (3) *Jacana spinosa* (L.); **Recurvirostridae**: (4) *Himantopus mexicanus* (Müller); **Laridae**: (4) *Leucophaeus atricilla* (L.), *Leucophaeus pipixcan* (Wagler); **Pelecaniformes: Ardeidae**: (4) *Nyctanassa violacea* (L.); **Threskiornithidae**: (2) *Platalea ajaja* (L.); (4) *Eudocimus albus* (L.); (4) *Plegadis chihi* (Viellot). **Passeriformes: Tyrannidae**: (4) *Pitangus sulphuratus* (L.); (4) *Tyrannus savana* (Daudin).

*Specimens deposited*: CNHE 12336–12340

*Representative DNA sequences*: PV700558–PV700572 (LSU); PV738124–PV738139 (*Nad1*).

**Figure 3.**
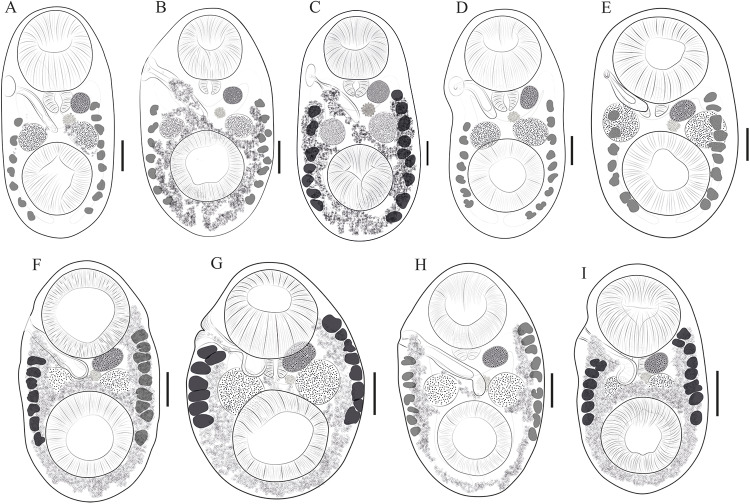
Drawings of *Stomylotrema* spp. from different hosts. (A–E) *Stomylotrema bijugum*; (A) *Eudocimus albus* from Tlacotalpan. (B) *Himantopus mexicanus* from Tlacotalpan. (C) *Leucophaeus atricilla* from La Polka. (D) *Leucophaeus pipixcan* from Tlacotalpan. (E) *Plegadis chihi* from Tlacotalpan. (F–I) *Stomylotrema vicarium*; (F) *Eudocimus albus* from Catemaco. (G) *Eudocimus albus* from Los Chivos. (H) *Himantopus mexicanus* from Tlacotalpan. (I) *Plegadis chihi* from Tlacotalpan. Scale bars A–I = 50 μm.

Redescription based on 21 specimens (Figure 3A–E). Comparative measurements from different hosts are provided in Table 2.

**Table 2.** Comparative measurements between adult specimens of *Stomylotrema bijugum* Braun, 1901 from different host species

				*Eudocimus albus*	*Plegadis chihi*	*Himantopus mexicanus*	*Leucophaeus atricilla*	*Leucophaeus pipixcan*
Host	*Himantopus mexicanus*	*Platalea ajaja*	*Jacana spinosa*	*n* = 5	*n* = 4	*n* = 6	*n* = 1	*n* = 5
Reference	Braun, 1901	Macko et al., 1999	CNHE 12081	**Present study**	**Present study**	**Present study**	**Present study**	**Present study**
Locality	Brazil	Playa Piloto, Cuba	Alvarado, Mexico	Tlacotalpan, Mexico	Tlacotalpan, Mexico	Tlacotalpan, Mexico	Tlacotalpan, Mexico	Tlacotalpan, Mexico
Infection site	**–**	Cloaca	Cloaca	Cloaca	Cloaca	Cloaca	Cloaca	Cloaca
Length (BL)	1300	2105–3213	1494	1360–1672 (1514)	1410–1470 (1431)	1716–1995 (1801)	1883	1480–1619 (1564)
Width (BW)	800	1708–2049	879	736–1116 (890)	827–893 (848)	960–1172 (1012)	991	675–826 (751)
Forebody (FB)	**–**	1160–1729 (1515)^a^	814	727–871 (804)	712–835 (764)	898–1072 (973)	1105	866–960 (915)
Hindbody (HB)	**–**	96–364 (240)^a^	251	154–195 (181)	123–141 (131)	218–302 (259)	247	163–185 (175)
Oral sucker (OSL × OSW)	323 × 323	679–869 × 805–890	413 × 428	441–597 (511) × 418–579 (499)	512–579 (532) × 503–545 (527)	502–589 (532) × 510–572 (533)	506 × 538	481–543 (503) × 452–552 (496)
Ventral sucker (VSL × VSW)	448 × 448	806–1027 × 930–1081	441 × 471	441–579 (494) × 434–750 (556)	474–515 (501) × 503–545 (527)	530–626 (572) × 495–651 (565)	540 × 546	415 × 565 (469)
Ratio (VSW/OSW)	**–**	1:1.1–1:1.2	1: 1.1	1:0.93–1.29 (1.10)	1:0.93–1.06 (1:0.99)	1:0.95–1.14 (1:1.06)	1: 1.01	1: 0.89–0.98 (1: 0.93)
Pharynx (PHL × PHW)	90 × 114	197–221 × 260–292	135 × 166	142–189 (159) × 168–186 (178)	189–206 (196) × 167–211 (188)	166–188 (178) × 169–198	168 × 211	144–172 (157) × 167–198 (182)
Ratio (OSL/PHL)	**–**	**–**	1: 3.09	1:2.9–3.89 (1:3.23)	1:2.56–2.79 (1:2.64)	1:2.84–3.55 (1:3)	1: 3.01	1:3.09–3.36 (1:3.21)
Testis poral (PTL × PTW)	**–**	324–466 × 371–454	206 × 210	148–267 (197) × 119–281 (190)	211–247 (228) × 229–265 (252)	206–231 (216) × 206–228 (218)	221 × 211	165–196 (181) × 158–241 (197)
Testis aporal (ATL × ATW)	**–**	324–466 × 371–454	297 × 214	152–274 (204) × 163–296 (215)	220–248 (229) × 241–284 (260)	206–231 (215) × 206–229 (219)	260 × 230	157–211 (183) × 182–250 (205)
Ovary (OLxOW)	**–**	158–324 × 269–324	146 × 154	95–176 (135) × 108–204 (148)	122–169 (147) × 157–200 (177)	146–176 (160) × 140–178 (166)	177 × 192	113–171 (139) × 124–156 (133)
Cirrus sac (CSL × CSW)	**–**	572–1104 (853) × 72–114 (86)^a^	492	358–528 (445) × 46–159 (96)	403–483 (435) × 92–108 (100)	436–627 (556) × 86–124 (104)	595 × 109	387–460 (414) × 67–93 (79)
Ratio (OSL/CSL)	**–**	**–**	1: 0.95	1:0.9–146 (1: 1.17)	1:1.05–1.27 (1.19)	1: 0.8–1.18 (1:0.98)	1:0.85	1:1.04–1.37 (1:1.22)
Ratio (CSW/OW)	**–**	–	1: 0.46	1:0.43–0.83 (1:0.57)	1:0.48–0.65 (1: 0.57)	1: 0.54–0.70 (1:0.63)	1:0.57	1:0.51–0.66 (1:0.6)
No. vitelline follicles poral	7	7	7	7	7	7	7	7
No. vitelline follicles aporal	9	9	9	9	9	9	9	9
Poral vitellaria extending to anterior part (DPV)	**–**	821–1178 (1031)^a^	595	616–604 (610)	603–672 (627)	605–1072 (759)	798	687–792 (730)
Aporal vitellaria extending to anterior part (DAV)	**–**	568–747 (684)^a^	481	464–563 (513)	492–603 (550)	521–857 (625)	705	565–654 (614)
Poral vitellaria extending to posterior part (DPP)	**–**	126–442 (280)^a^	188	89–216 (152)	102–234 (181)	223–353 (269)	309	166–226 (200)
Aporal vitellaria extending to posterior part (DAP)	**–**	221–347 (287)	247	35–43 (39)	52–194 (135)	156–404 (247)	180	102–220 (161)
Poral vitellaria field (FPV)	–	1031–1515 (1234)^a^	726	1018–1043 (1030)	633–665 (652)	729–933 (837)	882	618–763 (690)
Aporal vitellaria field (FAV)	–	1094–2000 (1572)^a^	782	761–1339 (1050)	650–954 (801)	801–1078 (957)	1109	817–901 (873)
Eggs (L × W)	19 × 14–18	27–33 × 15–19	–	19–33 × 14–22 (26–17)	–	26–32 × 15–19 (29–17)	22–31 × 12–20 (28–16)	–

Measurements are reported in micrometres (μm).

a Measurements from the original drawing.

Morphological identification: Digeneans 1360–1995 (1638) length and 675–1172 (918) wide. Tegument unspined. Subterminal oral sucker 441–589 × 452–572 (520 × 525). Ventral sucker 415–626 × 434–750 (523 × 548). Length ratio of oral and ventral sucker 1:0.89 to 1:1.29 (1:1.01). Pharynx 142–206 (174) × 167–211 (185). Caeca sometimes overlaps the lateral part of testes, terminating blindly beyond ventral sucker, almost reaching the posterior region of the body (Figure 3A–E). Testes equatorial anterior to ventral sucker and symmetrical. Poral testis 148–267 × 119–281 (217 × 224), aporal testis 152–274 × 163–296 (219 × 233). Cirrus sac straight or j-shape, reaching mid-body between testes, containing tubular coiled internal seminal vesicle (Figure 3A–E). Genital pore on the right margin of body located anterior to pharynx. Round ovary 95–177 × 108–204 (155 × 164), situated anterior to aporal testis (Figure 3A–E). Ovary larger than pharynx and in some cases the pharynx is almost the same size of ovary. Mehlis’ gland posterior to ovary. Seven poral and 9 aporal vitelline follicles, vitellaria slightly separated, situated laterally and overlapping caeca. Poral vitelline field commencing posterior to pharynx 602–1043 (781). Aporal vitelline field commencing to level or posterior to pharynx 650–1339 (913). Distance of the first poral vitellaria to the anterior end of the body 563–1072 (700). Distance of the last poral vitellaria to the posterior end 89–353 (218). Distance of the first aporal vitellaria to the anterior end of the body 464–857 (590). Distance of the last aporal vitellaria to the posterior end 35–404 (174). Both vitelline fields terminate near to posterior margin of ventral sucker (Figure 3A–E), exceptionally extending beyond it (Figure 3C). Uterus filling body surrounded or partially overlapping reproductive organs and ventral sucker. Eggs yellow, small 19–33 × 12–22 (26 × 16).

#### Remarks

Our specimens, collected from *E. albus, H. mexicanus, L. pipixcan* and *P. chihi* from 2 localities in Veracruz, and *L. atricilla* from Chiapas, were identified as *S. bijugum* by having features consistent with the diagnosis of the original description of Braun (1901) and the descriptions by Macko et al. (1999). The principal feature that distinguishes *S. bijugum* from other congeneric species is the horseshoe shape of the follicles and the termination of the vitelline fields near the posterior margin of ventral sucker. Additionally, our specimens showed great variability in features such as position, size and distribution of vitelline follicles, as well ovary and testes (Table 2).

### Stomylotrema vicarium *Braun, 1901*

*Site of infection*: Intestine and cloaca.

Metacercaria in coelom and gizzard.

Cercariae in the digestive gland.

*Type host*: (1) Pelecaniformes: Threskiornithidae: *Theristicus caerulenscens* (Vieillot).

*Type locality*: (1) Brazil (unspecified locality).

*Other localities*: Adult specimens records: (2, 4, 5) USA; (3, 6, 8) Argentina; (7) Cuba; (9) Peru; (10, 11) Mexico. Metacercarie specimens records: (12) Argentina; (13) Brazil. Cercariae specimen record: (14) Argentina.

Records: Adult specimens, 1. Braun (1901); 2. Lumsden and Zischke (1963); 3. Szidat (1964); 4. Bush and Forrester (1976); 5. Hon et al. (1978); 6. Ostrowski (1978); 7. Macko et al. (1999); 8. Lunaschi and Drago (2009); 9. Kanarek et al. (2017); 10. Ramírez-Cañas et al. (2019); 11. This study. Metacercariae specimens, 12. Ostrowski (1978); 13. Amato and Amato (2006). Cercariae, 14. Dellagnola et al. (2022).

*Other definitive hosts*: Class: **Mammalia. Didelphimorphia: Didelphidae**: (10) *Philander opossum* (L.); Class: **Aves. Galliformes: Phasianidae**: (6) *Gallus gallus domesticus* (L.); (5) *Meleagris gallopavo* (L.); **Podicipediformes: Podicipedidae**: (7) *Tachybaptus dominicus* (L.); **Accipitriformes: Accipitridae**: (8) *Busarellus nigricolis* (Latham); (8) *Buteogallus meridionalis* (Latham); **Charadriiformes: Recurvirostridae**: (11) *Himantopus mexicanus* (Müller); **Charadriidae**: (6) *Vanellus chilensis cayennensis* (Gmelin); **Laridae**: (3) *Larus dominicanus* (Lichtenstein); **Ciconiiformes: Ciconiidae**: (11) *Mycteria americana* (L.); **Pelecaniformes: Ardeidae**: (7) *Egretta caerulea* (L.), (2) *Nyctanassa violacea* (L.); **Threskiornithidae**: (4, 11) *Eudocimus albus* (L.); (11) *Plegadis chihi* (Viellot); **Passeriformes: Furnariidae**: (9) *Sclerurus mexicanus* (Sclater).

*Intermediate hosts*: (12) Insecta: Coleoptera: Dytiscidae: *Megadytes glaucus* (Brullé); (13) Hemiptera: Belostomatidae: *Belostoma dilatatum* (Dufour); (14) Gastropoda: Architaenioglossa: Ampullariidae: *Pomacea americanista* (Ihering).

*Specimens deposited*: CNHE 12341–12345

*Representative DNA sequences*: PV700573–PV700587 (LSU); PV738140–PV738147 (*Nad1*).

Description based on 53 specimens (Figure 3F–I). Measurements are provided in Table 3.

**Table 3.** Comparative measurements between adult specimens of *Stomylotrema vicarium* Braun, 1901 from different host species

					*Eudocimus albus*	*Plegadis chihi*	*Himantopus mexicanus*
Host	*Theristicus caerulescens*	*Egretta caerulea*	*Tachybaptus dominicus*	*Busarellus nigricolis*/*Buteogallus meridionalis*	n = 23	n = 18	n = 12
Reference	Braun 1901	Macko et al., 1999	Macko et al., 1999	Lunaschi and Drago, 2009	**Present study**	**Present study**	**Present study**
Locality	Brazil	Maria la Gorda, Cuba	Guanahacabibes Lake Reservation, Cuba	La Marcela/Pirané Argentina	Tlacotalpan, Mexico	Tlacotalpan, Mexico	Tlacotalpan, Mexico
Infection site	–	Cloaca	Cloaca	Cloaca	Cloaca/Intestine	Cloaca/Intestine	Cloaca/Intestine
Length (BL)	2100	2041–2417	2835–3315	1700–2500 (2100)	685–1989 (1134)	674–1472 (958)	1031–1306 (1190)
Width (BW)	1300	1550–1663	2022–2381	1200–1300 (1300)	389–1046 (692)	420–1002 (568)	644–865 (733)
Forebody (FB)	–	1137–1394 (1265)^a^	1559–2018 (1788)^a^	2951^a^	405–1131 (658)	391–1022 (563)	586–756 (692)
Hindbody (HB)	–	137–201 (169)^a^	366–385 (375)^a^	585^a^	67–239 (103)	57–166 (90)	56–130 (106)
Oral sucker (OSL × OSW)	573 × 625	672–742 × 719–801	853–869 × 853–880	532–619 (577) × 600–725 (674)	209–594 (397) × 216–573 (403)	222–551 (328) × 214–597 (333)	382–454 (414) × 399–452 (423)
Ventral sucker (VSL × VSW)	625 × 647	687–751 × 742–828	948–969 × 948–964	696–870(774) × 764–861(812)	206–646 (375) × 179–536 (401)	208–597 (317) × 215–587 (320)	327–442 (397) × 359–464 (410)
Ratio (VSW/OSW)	–	1:1.09–1:1.13	1:1.09–1:1.13	1: 1.1–1.4 (1:1.2)	1:0.83–1.08 (1: 0.98)	1: 0.85–1.04 (1:0.95)	1:0.89–1.04 (1:0.97)
Pharynx (PHL × PHW)	156 × 187	190–205 × 213–258	237–269 × 240–272	135–193 (158) × 193–203 (200)	71–188 (124) × 100–195 (141)	80–181 (108) × 95–183 (128)	116–161 (134) × 130–150 (144)
Ratio (OSL/PHL)	–	–	–	1:3.2–4.0 (1:3.7)	1: 2.6–3.85 (1:3.24)	1:2.74–3.68 (1:3.0)	1: 2.6–3.47 (1:3.10)
Testis poral (PTL × PTW)	–	316–426 × 371–466	411–521 × 411–592	208–232 (222) × 232–290 (254)	74–234 (153) × 64–260 (162)	51–299 (122) × 62–290 (133)	145–218 (185) × 175–218 (203)
Testis aporal (ATL × ATW)	–	316–426 × 371–466	411–521 × 411–592	193–227 (208) × 217–256 (238)	73–224 (155) × 83–257 (172)	62–299 (128) × 66–320 (139)	143–231 (189) × 176–233 (208)
Ovary (OL × OW)	–	198–213 × 198–233	298–371 × 324–411	155–217 (184) × 193–232 (214)	50–149 (104) × 48–186 (118)	44–184 (87) × 40–227 (91)	109–141 (121) × 124–165 (145)
Cirrus sac (CSL × CSW)	–	734–853 × 146–182	948–1106 × 182–237	401–555 (469) × 121–126 (124)	174–560 (314) × 44–109 (73)	176–610 (272) × 31–114 (57)	383–491 (418) × 78
Ratio (OSL/CSL)	–	–	–	1:1.1–1.3 (1:1.2)	1:0.90–1.72 (1:1.30)	1:0.90–1.6 (1:1.22)	1:0.86–1.10 (1:1)
Ratio (CSW/OW)	–	–	–	–	1:0.48–1.02 (0.68)	1:0.37–1.15 (1:0.7)	1:0.51–0.74 (1:0.61)
No. vitelline follicles poral	7	7	7	7	7	7	7
No. vitelline follicles aporal	9	9	9	9	9	9	9
Poral vitellaria extending to anterior part (DPV)	–	559–743 (651)^a^	977–1095 (1036)^a^	710^a^	290–842 (443)	287–627 (402)	421–512 (464)
Aporal vitellaria extending to anterior part (DAV)	–	394–889 (641)^a^	323–823 (573)^a^	613^a^	178–739 (330)	219–569 (301)	314–361 (336)
Poral vitellaria extending to posterior part (DPP)	–	458–550 (504)^a^	669–867 (768)^a^	484^a^	230–383 (284)	136–450 (239)	205–422 (302)
Aporal vitellaria extending to posterior part (DAP)	–	458–633 (545)^a^	691–860 (775)^a^	606^a^	209–343 (276)	52–316 (220)	227–344 (291)
Poral vitellaria field (FPV)	–	871–1100 (985)^a^	1147–1404 (1275)^a^	1000^a^	116–983 (418)	210–633 (330)	335–536 (431)
Aporal vitellaria field (FAV)	–	853–1018 (935)^a^	1529–1683 (1606)^a^	1161^a^	100–812 (465)	246–954 (418)	422–694 (568)
Eggs (L × W)	26–29 × 14–17	31–34 × 17–20	27–34 × 17–24	26–29 × 14–17 (27 × 15)	21–30 × 11–17 (26 × 14)	24–29 × 13–17 (26–15)	23–30 × 13–20 (26 × 15)

Measurements are reported in micrometres (μm).

a Measurements from the original drawing.

Morphological identification: Digeneans 862–1989 (1178) length and 534–1002 (726) wide. Tegument, unspined. Subterminal oral sucker 300–594 × 320–597 (419 × 430). Ventral sucker 288–646 × 303–587 (402 × 423). Length ratio of oral and ventral sucker 1:0.89–1.08 (1:0.98). Pharynx 83–181 × 115–195 (131 × 145). Caeca overlapping lateral part of testes, terminating posterior to ventral sucker (Figure 2F–I). Round or oval testes are symmetrical, anterior to ventral sucker. Poral testis 99–299 × 110–290 (175 × 188), aporal testis 107–299 × 116–320 (178 × 196). Cirrus sac straight or j-shape, reaching mid-body between testes and in some specimens, it ends before the poral testis, containing tubular coiled internal seminal vesicle (Figure 3F–I). Genital pore on right margin or submarginal of body located posterior to distal half of oral sucker. Round ovary 69–184 × 75–227 (116 × 136), situated anterior to aporal testis (Figure 3F–I). Mehlis’ gland posterior to ovary. Seven poral and 9 aporal vitelline follicles, vitellaria compact, situated laterally and marginal. Poral vitelline field commencing at level of the pharynx 116–983 (425). Aporal field commencing at mid-part of oral sucker 71–954 (533). Distance of the first poral vitellaria to the anterior end of the body 324–842 (459). Distance of the last poral vitellaria to the posterior end 205–450 (296). Distance of the first aporal vitellaria to the anterior end of the body 178–739 (336). Distance of the last aporal vitellaria to the posterior end 52–344 (280). Both vitelline fields terminate laterally to the mid-part of the ventral sucker (Figure 3F–I). Uterus filling body and winding around the ventral sucker. Eggs yellow, small and oval 21–30 × 11–20 (26 × 15).

#### Remarks

The specimens collected from *E. albus, P. chihi* and *H. mexicanus* from 3 localities in Veracruz were identified as *S. vicarium* by having features consistent with the original description by Braun (1901). The principal feature that distinguishes to *S. vicarium* from other congeneric species is the lateral and linear position of the vitelline fields.

### Statistical analyses

**Figure 4.**
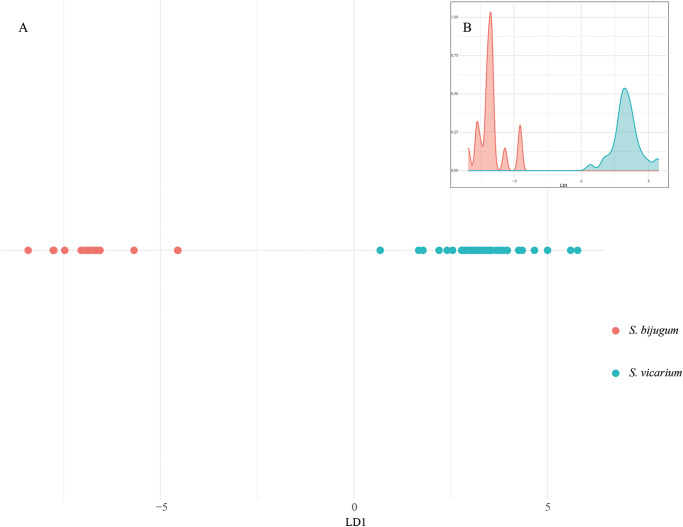
Statistical analyses. Discriminant analysis (A); linear discriminant analysis (B); density distribution of LD1. The colours represent the species of *Stomylotrema* spp. recovered: in red *S. bijugum*, in blue *S. vicarium.*

Based on 32 morphological measurements, the LDA was performed to evaluate the morphological differentiation between *Stomylotrema bijugum* and *S. vicarium*. The coefficients of the discriminant functions indicate the contribution of each variable to the separation between the species. LD1 explains 100% of the variance, which indicates that all relevant and useful information to differentiate species is contained in a single dimension. Morphometric ratios, such as the relationship between R(OSW/PHW), R(MTW/OW), R(CSW/OW) and R(OSL/CSL), contributed the most to the separation between the species in LD1 (Figure 4A). In addition, the density distribution of LD1 values shows a clear separation between the 2 species, with *S. bijugum* (in red colour) clustering at negative values and *S. vicarium* (in blue colour) at positive values (Figure 4B), and the lack of significant overlap between the density distributions was significant, and confirms that LDA effectively differentiates these species based on morphological traits (Wilks’ Lambda = 0.045; *F* = 13.75, *P* < 0.0001).

**Figure 5.**
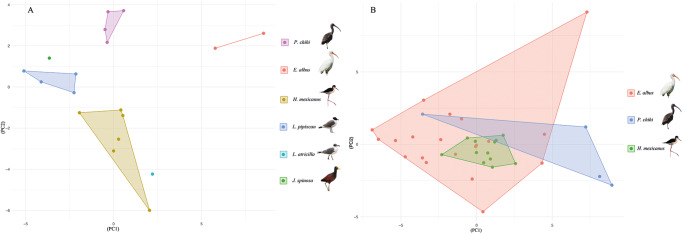
Principal component analysis conducted with 32 morphometric variabilities from 54 specimens of *Stomylotrema* spp., analysed by host species (A) *S. bijugum* and (B) *S. vicarium.*

A PCA was conducted to explore the morphological differences among the isolates of *S. bijugum* and *S. vicarium* (Figure 5A and B). The measurements of specimens of *S. bijugum* from 6 different host species formed 3 not overlapping polygons corresponding to the 3 host species (*P. chihi, H. mexicanus* and *L. pipixcan*) addressed on this study. However, the specimens from the remaining 3 hosts (*E. albus, J. spinosa* and *L. atricilla*) did not form polygons due to the limited number of measurements (*n* < 3) available per host species. The first and second components explain 34.21% and 23.51% (57.72% accumulative) of the variance, respectively (Figure 5A). Conversely, the measurements of the specimens of *S. vicarium* from 3 different host species formed 3 polygons overlapped with each other. The first and second components explain 47.39% and 14.47% (61.86% accumulative) of the variance, respectively (Figure 5B).

### Phylogenetic analysis and haplotype network

**Figure 6.**
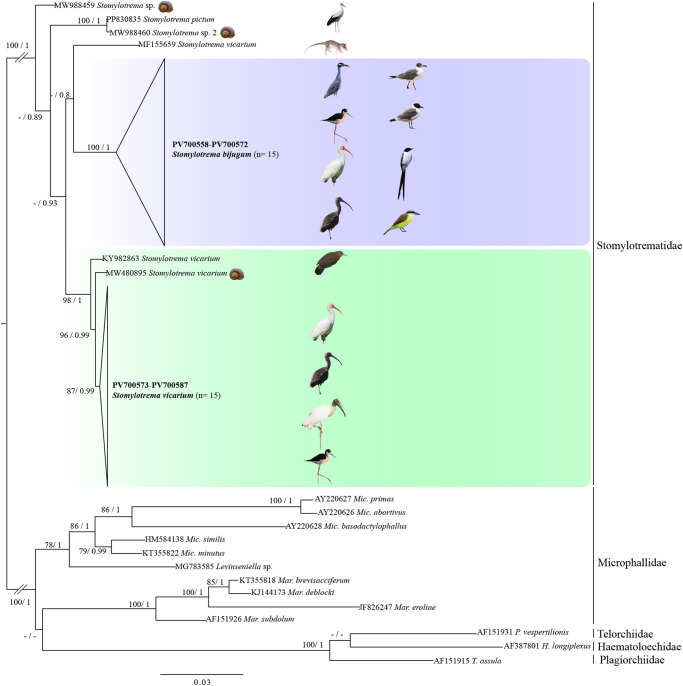
Phylogenetic trees inferred with maximum likelihood (ML) and consensus Bayesian inference (BI) of LSU from nuclear ribosomal DNA. Numbers near internal nodes show maximum likelihood bootstrap percentage values and Bayesian posterior probabilities. Sequences generated in this study are in bold.

Phylogenetic analyses inferred with ML and IB showed that all sequences of *Stomylotrema* formed a clade with strong bootstrap support and Bayesian posterior probability values (Figure 6). All the sequences obtained in this study formed 2 independent subclades. The first one was formed with 15 newly sequences identified as *S. bijugum*, recovered from 8 host species (*E. albus, H. mexicanus, L. atricilla, L. pipixcan, N. violacea, P. chihi, P. sulphuratus* and *T. savana* from Tlacotalpan, Veracruz and La Polka, Chiapas, Mexico) and its sister species was *S. vicarium* (MF155659) from the grey 4-eyed opossum (*Philander opossum* L.) from Mexico with a weak support of bootstrap and Bayesian posterior probabilities.

The second subclade was formed with 16 newly sequences identified as *S. vicarium* from 4 host species (*E. albus, H. mexicanus, M. americana* and *P. chihi* from Tlacotalpan, Veracruz, Mexico), plus 2 sequences identified as *S. vicarium* from Peru (KY982863) and Argentina (MW480895). Two unidentified isolates of *Stomylotrema* sp. (MW988459-460) formed 2 independent subclades. An isolate identified as *Stomylotrema* sp. 2 (MW988460) from Egypt is sister to an isolate identified as *S. pictum* (PP830835) from Turkey (Figure 6). The genetic divergence estimated with the LSU among the species of *Stomylotrema* ranged from 2.2% to 4.8%, and among the newly sequences identified as *S. bijugum* and *S. vicarium* ranged from 2.6 to 4.2%. In addition, the genetic distances among our isolates of *S. vicarium* and *S. vicarium* available in GenBank (MW480895 and KY982863) ranged from 0.3% to 1.1% and between our isolates of *S. vicarium* and *S. vicarium* (MF155659) from the grey 4-eyed opossum (*P. opossum*) ranged 3.3% to 3.9%. Finally, among the newly sequences of *S. bijugum* and *S. vicarium* (MW480895 and KY982863) ranged from 2.7% to 3.2%, *S. vicarium* (MF155659) ranged from 3.6% to 4.8%. The intraspecific genetic divergence ranged 0% to 1.4% on *S. vicarium* and from 0% to 1.1% on *S. bijugum* (Table 4).

**Table 4.** Genetic divergence estimated among the species of *Stomylotrema* with the large subunit of the nuclear ribosomal DNA

	MW988459 *Stomylotrema* sp. 1	MW988460 *Stomylotrema* sp. 2	PP830835 *Stomylotrema pictum*	MF155659 *Stomylotrema vicarium*	*Stomylotrema bijugum*	KY982863 *Stomylotrema vicarium*	MW480895 *Stomylotrema vicarium*	*Stomylotrema vicarium*
MW988459 *Stomylotrema* sp. 1	–							
MW988460 *Stomylotrema* sp. 2	2.9	–						
PP830835 *Stomylotrema pictum*	3.06	0.09	–					
MF155659 *Stomylotrema vicarium*	3.8	4.7	4.73	–				
*Stomylotrema bijugum* (*n* = 15)	2.2–3.3	3.5	4	3.6–4.8	0–1.4			
KY982863 *Stomylotrema vicarium*	2.8	3.4	3.5	3.7	2.7–3.1	–		
MW480895 *Stomylotrema vicarium*	3	3.4	3.6	3.4	2.8–3.2	1	–	
*Stomylotrema vicarium* (*n* = 16)	2.6–3.3	3.2	3.8	3.3–3.9	2.6–4.2	0.74–1.1	0.3–0.7	0–0.4

Uncorrected *p* distances are expressed as percentages.

**Figure 7.**
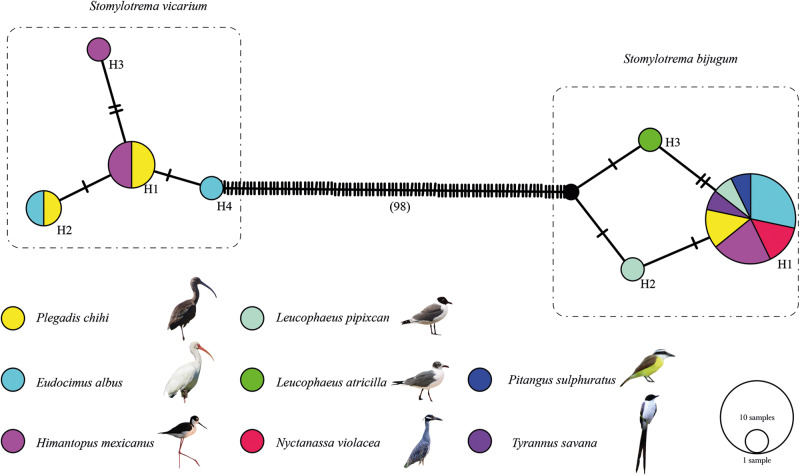
Haplotype network of *Stomylotrema* spp., built with the gene nicotinamide adenine dinucleotide dehydrogenase subunit 1 (*Nad1*). Each circle represents a haplotype, with size proportional to the haplotype´s frequency.

The haplotype network built in this study was inferred with 24 sequences and 449 characters. The haplotype network yielded 2 subgroups representing *S. vicarium* and *S. bijugum*, clearly separated from each other by 98 substitutions. The first subgroup contained 8 specimens of *S. vicarium* with 4 haplotypes separated each other by a few substitutions and were shared among 3 definitive hosts sampled. The most frequent haplotype (H1, *n* = 4) corresponded to specimens from 2 hosts (*P. chihi* and *H. mexicanus*). The second subgroup contained 16 specimens of *S. bijugum* with 3 haplotypes separated each other by 1 or 2 substitutions. The most frequent haplotype (H1, *n* = 14) corresponded to specimens from 7 definitive hosts sampled (*E. albus, H. mexicanus, N. violacea, P. chihi, P. sulphuratus, T. savana* and *L. pipixcan*) (Figure 7).

## Discussion

The present study provides morphological, morphometric, molecular and ecological evidence of 2 congeneric *Stomylotrema* species. Adult specimens of *S. bijugum* and *S. vicarium* were recorded for the first time in aquatic and passerine birds from southeastern Mexico, revealing new hosts and geographical areas in the Americas. Our morphological observations revealed that *S. bijugum* can be distinguished from *S. vicarium* in terms of the position of the vitelline follicles. In *S. bijugum*, the vitellin follicles start posterior to the genital pore, reach the posterior part of the ventral sucker and end in the posterior end of the body, whereas vitellin follicles start anterior-posterior to the genital pore and end in the middle part of the ventral sucker in *S. vicarium.*

In addition, the LDA clearly revealed that 6 morphometric variables (the ratio between the suckers, pharynx, ovary and cirrus sac) were able to discriminate between the 2 species. The species *S. bijugum* was recorded from the Black Wing (*Himantopus himantopus*) from Brazil and later from the Roseate Spoonbill (*Platalea ajaja*) in Cuba (Macko et al., 1999). In the present study, *S. bijugum* was recorded from 5 host species in southeastern Mexico. Adult specimens were measured and compared with previous records, and the PCA clearly revealed 3 independent polygons corresponding to the specimens from the 3 host species from southeastern Mexico, suggesting host-induced phenotypic plasticity.

The species *S. vicarium* was recorded from plumbeous ibis (*Theristicus caerulenscens*) in Brazil and later from 5 host species in the USA, Argentina and Cuba (Macko et al., 1999). Ostrowski (1978) and Macko et al. (1999) noted that *S. vicarium* is a species with a high level of morphological variability caused by the age of the parasite. In the present study, adult specimens of *S. vicarium* from 3 host species were evaluated and compared with previous descriptions. Our observations and morphometric data revealed morphological differences among the specimens sampled from the 3 host species. The PCAs revealed 3 clustered together, suggesting that those specimens were morphometrically homogeneous.

The application of molecular analyses to distinguish species of the genus *Stomylotrema* has rarely been addressed, but it is key to the delineation of the species. Therefore, in this study, sequences of *Nad1* from the mitochondrial gene were generated and analysed. The haplotype network analysis of *Nad1* sequences predicted with 25 sequences revealed the presence of 2 clusters belonging to *S. bijugum* and *S. vicarium*, which were separated from each other by 98 substitutions, confirming that both clusters belong to 2 species. In addition, our phylogenetic analyses inferred with the LSU sequence from nuclear rDNA confirmed that our specimens identified as *S. bijugum* and *S. vicarium* formed 2 independent subclades. The phylogeny presented here has suggested that 2 isolates identified as *S. vicarium* whose sequences are available in GenBank (MW480895 and KY982863) from Argentina and Peru, respectively, were placed in a clade together with our *S. vicarium* specimens, confirming that all the specimens are conspecific. Another isolate from the grey 4-eyed opossum (*Philander opossum* L.) in Mexicoidentified as *S. vicarium* available in GenBank (MF155659) represents in this study a lineage independent of both *S. vicarium* and *S. bijugum*, suggesting that this isolate may correspond to a new species. However, this specimen was not deposited in any collection, and the report could not be verified.

The level of genetic divergence found among the *Stomylotrema* species could provide additional evidence for the delineation of these species. The intraspecific genetic divergence among 15 isolates of *S. bijugum* was very low, ranging from 0% to 1.4%; among the 16 isolates of *S. vicarium*, the intraspecific genetic divergence ranged from 0% to 0.4%; among our isolates of *S. vicarium* and *S. vicarium* available in GenBank (MW480895 and KY982863), the intraspecific genetic divergence ranged from 0.3% to 1.1%; between our isolates of *S. vicarium* and *S. vicarium* (MF155659) and the specimen isolated from the grey 4-eyed opossum (*P. opossum*), the intraspecific genetic divergence ranged from 3.3% to 3.9%; and between the new sequences of *S. bijugum* and *S. vicarium*, the intraspecific genetic divergence ranged from 2.6% to 4.2%. The percentage of interspecific genetic divergence found is similar to that reported in other species of the family Microphallidae (sister to Stomylotrematidae), ranging from 1.0% to 9.3%, from 5.25% to 7.92% and from 1.5% to 3.3% among species of *Maritrema* (Presswell et al., 2014; Hernández-Orts et al., 2020; Aldama-Prieto et al., 2024), or among species of *Microphallus*, ranging from 1.1% to 7% (Galaktionov and Blasco-Costa, 2018) and from 6.5% to 11 % (Kudlai et al., 2015).

Ecological evidence suggests that *S. bijugum* and *S. vicarium* have low host specificity and may have a wide range of definitive hosts, facilitating their dispersion and distribution in the Americas. The life cycle of *S. bijugum* is unknown. However, the life cycle of *S. vicarium* was recently characterized by combining morphological and molecular data. Dellagnola et al. (2022) reported that the apple snail (*Pamacea americanista*) serves as the first intermediate host and that invertebrates, such as the coleopter (*Megadytes glaucus*) and hemipter (*Belostoma dilatatum*), serve as second intermediate hosts, which are eaten by several birds that serve as definitive hosts (Ostrowski, 1978; Amato and Amato, 2006), and that mammals also serve as definitive hosts of species of *Stomylotrema* (Ramírez-Cañas et al., 2019). In this study, birds from the families Ardeidae, Laridae, Threskiornithidae and Tyrannidae were recorded as definitive hosts to *S. bijugum* and *S. vicarium*, suggesting that the life cycle of both species addressed in this study can be completed in southeastern Mexico.
